# Safety in Wine Production: A Pilot Study on the Quality Evaluation of *Prosecco* Wine in the Framework of UE Regulation

**DOI:** 10.3390/ijerph17093283

**Published:** 2020-05-08

**Authors:** Vincenzo Marcotrigiano, Sandro Cinquetti, Riccardo Flamini, Mirko De Rosso, Luca Ferraro, Saverio Petrilli, Matilde Poggi, Alessandro Dettori, Anna De Polo, Osvalda De Giglio, Giovanni Battista Orsi, Maria Teresa Montagna, Christian Napoli

**Affiliations:** 1 Food Hygiene and Nutrition Service, Department of Prevention, Local Health Unit BT, Corso M. R. Imbriani 138, 76125 Trani, Barletta-Andria-Trani, Italy; 2 Hygiene and Public Health Service, Department of Prevention, Local Health Unit 2 Marca Trevigiana, Via Sant’Ambrogio di Fiera 37, 31100 Treviso, Italy; sandro.cinquetti@aulss2.veneto.it (S.C.); anna.depolo@aulss2.veneto.it (A.D.P.); 3 Council for Agricultural Research and Economics—Viticulture & Enology (CREA-VE), Viale XXVIII Aprile 26, 31015 Conegliano, Treviso, Italy; riccardo.flamini@crea.gov.it (R.F.); mirko.derosso@crea.gov.it (M.D.R.); 4 Italian Federation of Independent Winemakers—FIVI, Località La Croix-Noire—Rue Croix-Noire, 76 Saint Christophe, 11100 Aosta, Italy; luca@belecasel.it (L.F.); saverio@valgiano.it (S.P.); mpoggi@fraghe.it (M.P.); alessandro@tenutedettori.it (A.D.); 5 School of Specialization in Hygiene, Preventive Medicine and Public Health, University of Padua, Via Giustiniani 2, 35128 Padua, Italy; 6 Department of Biomedical Sciences and Human Oncology, Section of Hygiene, University of Bari Aldo Moro, Medical School, Piazza G. Cesare 11, 70124 Bari, Italy; osvalda.degiglio@uniba.it (O.D.G.); mariateresa.montagna@uniba.it (M.T.M.); 7 Department of Public Health and Infectious Diseases, “Sapienza” University of Rome, Piazzale Aldo Moro 5, 00185 Rome, Italy; giovanni.orsi@uniroma1.it; 8 Department of Medical Surgical Sciences and Translational Medicine, “Sapienza” University of Rome, Via di Grottarossa 1035/1039, 00189 Rome, Italy; christian.napoli@uniroma1.it

**Keywords:** risk assessment, food safety, wine production, plant protection products

## Abstract

In Italy, wine production is considered a sector of excellence, where the wines’ appreciable sensory features are favored by environmental factors, including weather and climate conditions, which benefit territories with a specific vocation. The whole chain involves many economic and agri-food sector operators, and requires an in-depth assessment of specific risks for identifying critical points, keeping the entire production process under control, and ensuring product traceability. This article describes the results of a pilot study conducted in the Prosecco DOCG (Designations of Controlled and Guaranteed Origin) area, concerning the detection of residues of plant protection products in fifty wine bottles. Although considerably below the maximum residue levels, all the samples tested were positive, ranging from two to five active substances detected in each sample. In addition to the provisions of the European Community legislation, this paper critically evaluates some best practices models that are already used by the Wine Federations of Italy, with the aim of identifying advantages of and areas for improvement in production methods, applicable to raw materials reception, rasping, storage, and bottling phases, in order to guarantee product safety and quality.

## 1. Introduction

Wine production represents a sector of excellence in Italy, where the wines’ appreciable features are favored by the climatic conditions of specific territories. The quality of raw materials is a fundamental requirement to obtain the best-quality product, which is frequently exported to both European Union (EU) and non-EU countries.

The most recent Italian Ministry of Agricultural, Food and Forestry Policies data (MIPAAF) reports the production of more than 53 million hectoliters of wine in Italian wine-producing factories, of which about 14 million hectoliters are represented by Protected Geographical Indications (IGP) wines and almost 27 million hectoliters by Protected Denomination of Origin (DOP) wines [1].

The variable geomorphological features of our territory and its specific conformations (mountain, hills, or coastal) favor the cultivation of several vineyard typologies. Besides internationally well-known vineyards such as Nebbiolo, Sangiovese, Primitivo and Prosecco, there are hundreds of new and rapidly rising commercial products, such as Fumin, Arneis, Vermentino, Cannonau, Nero d’Avola, Gaglioppo, Negramaro, Fiano, Tintilia, Montepulciano, Verdicchio, Cesanese, Trebbiano, Albana, Croatina, Garganega, Ribolla, Nosiola and Lagrein.

Wine is produced within a wide system of protected territories, managed by “Protection Consortia”, which represent local wine production stakeholders. It is organized by the specifications of origin designation, which also gathers the Designations of Controlled and Guaranteed Origin (DOCG). These designations promote the conservation of traditional practices as well as innovation, in delimited areas. Moreover, chemical and organoleptic analyses are required to obtain and maintain the designation of origin, in order to commercialize a more guaranteed product. Currently, in Italy, 64% of wine production is commercialized with a designation of origin.

### 1.1. Food Chain and Risk Assessment

Wine is the product of alcoholic fermentation operated by yeasts on the grape skin. Its juice turns from sugary into alcoholic liquid through chemical reactions. In the winemaking process, the “winemaker” represents the junction point between territories and consumers. This figure is responsible for both checking the production chain and, sometimes, for direct sales that reduce commercial passages.

The production chain also involves several economic and agri-food sector operators (ASOs), from field production to transformation and bottling for the subsequent distribution and consumption. Nevertheless, such a wide food chain requires a deep and specific risk assessment, which is useful to identify critical points, control the entire production process, and to guarantee the traceability of the final product [2].

All food industries must comply with general and specific requirements and notify to the competent territorial authority about their activities, for example, in other food sectors [3,4,5]. The sector’s requirements are applicable to all the steps from receiving raw materials to destemming, storing, and bottling.

The risk coming from biological agents can be reasonably considered negligible in the final products, given that potentially pathogenic microorganisms can hardly survive at low pH values and with the alcohol content of wine. On the contrary, the exposure to chemical risks attributable to the possible presence of heavy metals, sulfur dioxide, and residues of plant protection products (PPPs) must be considered. The use of chemical pesticides has been a standard practice in European conventional agriculture, increasing the yields of agricultural crops by protecting them against weeds, pests, and diseases [6,7]. However, pesticides can also have undesirable effects on human health and on the environment [8], which is a major issue of concern for public health [9].

For this reason, sector studies have investigated the presence of contaminants (e.g., heavy metals, mycotoxins, sulfite residues) in wines produced in the EU, belonging to both conventional and organic circuits. Although in the most representative studies there were no significant differences regarding the content of sulfites and ochratoxin A in the two different types of products, in organic wines the presence of lead and magnesium was detected in lower quantities than in conventional wines [10,11].

Food products made with traditional techniques, according to the adoption of codified specifications, are protected by specific EU legislation, especially in a world characterized by emerging and re-emerging diseases [12,13,14]. However, member states may apply local additional regulations to establishments in which foods are produced with traditional characteristics. It is the responsibility of the ASO to ensure that the food meets the provisions of food law at all stages of production, processing, and distribution. Some examples of best practices are already in use and promoted by industry federations, which encourage the use of ASO food safety plans according to a controlled production cycle [15].

### 1.2. Organic or Conventional Products

Although many types of active substances are currently marketed and used, including fungicides, acaricides, insecticides, and herbicides, the fight against pests is also carried out with natural products [16]. In general, weed management under organic agriculture practices is complicated because the available natural herbicides have little or no selectivity and they must be applied in relatively large quantities. This may lead to undesirable effects on the environment and the soil fauna and microbes, failing the principles of organic agriculture [17].

In the first half of the 19th century, extracts from *Pyrethrum* flowers made their debut as organic household insecticides [18], and shortly after that, the nicotine, extracted from tobacco plants, was used as an organic pesticide in crop protection [19]. After 1930 a broad search for new organic and chemical crop protection agents began.

With regard to grapevine protection, some examples were the fight against downy mildew and powdery mildew of grapevine, brought to Europe in 1878 and 1845, respectively. Downy mildew spread quickly, causing damage to vineyards until 1885, when the French botanic professors Pierre Alexis Millardet and Ulysse Gayon discovered the effect of Bordeaux mixture [20]. Since then, copper has been an irreplaceable agent in the suppression of downy mildew of grapevine. Powdery mildew caused great damage to vineyards until sulfur was found to be efficient in its suppression [21]. Moreover, arsenite has also been applied for a long time in France to suppress the agents causing grapevine wood disease, but its use has been prohibited since 2001 [22]. Finally, in 2000, the European and Mediterranean organization for plant protection established the standard for the best practices regarding grapevine protection by organic production [23]. The list of the allowed pesticides for organic production was updated in the EU up to 2012 [24,25].

The corroborating substances, coming from natural origin elements, are useful to improve the resistance of plants against harmful organisms and to protect them from damage not caused by pests. These substances are used in organic agriculture as enhancers of plant defenses. Some examples include biodynamic preparations, stone or rock powder, sodium bicarbonate, silica gel, edible vegetable oils (peanut, safflower, cotton, sunflower, flax, corn, etc.), lecithin, and vinegar. Products containing corroborating substances are not subject to authorization for placement on the market since their use does not cause harmful effects (neither immediate nor delayed) on humans, animals, or the environment. The MIPAAF periodically updates the list of corroborants, prescribing reports on the label indications regarding the qualitative/quantitative composition, modalities, intended use, and precautions for use.

On the other hand, PPPs are products containing active chemical substances, agronomic antidotes or synergists intended to protect plants or plant products from all harmful organisms or prevent the effects of the latter. Moreover, PPPs are useful to influence the vital processes of plants, preserve plant products, or destroy unwanted plants or parts of them [26]. These products are commonly synthesized and require authorization for placement on the market, and are classified according to the degree of toxicity and are labelled with risk indications and safety advice. The toxicological classification provides categories of PPP belonging to different hazard classes. The most recent ISTAT data show that more than 126,000 tons of PPPs for agricultural use were marketed in Italy in 2018, comprising approximately 59,000 tons of fungicides, 23,000 tons of insecticides and acaricides, 24,000 tons of herbicides, and 20,000 tons of various active ingredients [27].

Any person who purchases, transports, or distributes PPPs must have a specific authorization, obtained by successfully passing a training course. In this way, adequate knowledge of the risks coming from managing PPPs is guaranteed, with particular attention to compliance with the safety interval (commonly a shortage time)—that is, the minimum time period that must elapse between treatment and the harvest of agricultural products.

As part of Italian wine products, Prosecco DOCG is a high-quality wine produced in the mountain foothills of Veneto Region (north-east Italy). Grapes are produced by conventional viticulture following a restrictive protocol—Conegliano-Valdobbiadene Prosecco Superiore DOCG Viticulture Protocol—which allows the use of some pesticides, but not those suspected to be carcinogenic, such as dithiocarbamates (e.g., mancozeb) and folpet [28]. In this viticulture protocol, some active substances are allowed “just for limited use”: abamectin B1, benzoximate, chlorpyrifos, chlorpyrifos-methyl, cymoxanil, cyprodinil, dimethomorph, indoxacarb, metalaxyl, pyraclostrobin, pyridaben, spiroxamine, tebufenpyrad, trifloxystrobin, and zoxamide.

Reference laboratories use standard analytical panels in order to process food matrices sampled by Health Authorities during official routine controls. These panels are standard for food matrices of plant origin in general and are not food-specific; therefore, it could happen that food-specific active substances are not completely investigated by standard panels. In the case of wine, only 13 active substances of those included in the Prosecco Viticulture Protocol are investigated during official routine controls; while the other two (abamectin B1, benzoximate) and two mancozeb and folpet metabolites are not included in the standard routine panel. In order to fill this gap, it is necessary to deepen the research, widening the standard analytical panel for this specific food matrix, including suspected carcinogenic substances.

In this context, the objective of the present study was to investigate the presence of the abovementioned 15 active substances allowed “just for limited use”, and two folpet and mancozeb metabolites (phthalimide and ethylene thiourea, respectively) in samples of Prosecco wines coming from the geographical Conegliano-Valdobbiadene Prosecco DOCG area.

## 2. Materials and Methods

During the year 2016, fifty bottles of Prosecco wine were randomly selected from about 210 wine industries located in Conegliano-Valdobbiadene Prosecco DOCG area, and tested for the ingredients allowed “just for limited use”, as well as the abovementioned metabolites. Analyses were performed according to the method by Payá et al., modified for our aims [29].

A 3 mL wine sample was diluted by the addition of 1 mL acetonitrile, then 2 g NaCl and 1 g Na_2_SO_4_ were added, keeping the solution under stirring for 1.5 min. After centrifugation, 0.3 mL of the upper phase was recovered, and the solution was diluted by water 1:1 (*v/v*) and filtered through an Agilent Captiva 0.22 µm nylon filter (Agilent Technologies, Santa Clara, CA, USA).

Analysis was performed using an Agilent 1290 Infinity ultra-high performance liquid chromatograph (UHPLC) coupled to an Agilent 1290 Infinity Autosampler (G4226A) and an Agilent 6540 accurate-mass quadrupole-time of flight (Q-TOF) mass spectrometer (nominal resolution 40.000) with a Dual Agilent Jet Stream Ionization source (Agilent Technologies, Santa Clara, CA, USA).

Chromatographic separation was performed using a Zorbax SB C_18_ (100 mm × 2.1 mm; 1.8 µm) column (Agilent Technologies, Santa Clara, CA, USA) and mobile phase composed of: (A) 10% acetonitrile/90% water pH 6.00 and (B) 90% acetonitrile/10% ammonium formate buffer pH 6.00 (flow rate 0.25 mL/min).

Standard compounds were purchased by Fluka (Merck Life Science S.r.l., Milano, Italy). Standard solutions were prepared using an organic wine in which the absence of residues was previously checked. The wine was spiked with standard compounds at concentrations between 0.009 and 1 mg/L.

For the quantification of the residues in the samples, the following calibration curves were used: cyprodinil y = 1.667 x −0.034, R² = 0.999; dimethomorph y = 5.919 x −0.059, R² = 0.999; metalaxyl y = 8.102 x +0.125, R² = 0.999; zoxamide: y = 5.053 x +0.117; R² = 0.999.

For phthalimide analysis, solid-phase extraction (SPE) sample preparation was performed. Two milliliters of sample diluted 4-fold by water was passed through a 360 mg C_18_ cartridge (Waters). Stationary phase was washed with 2 mL water, then the analyte was recovered with 3 mL of dichloromethane. Solution was brought to dryness by rotavapor, the residue dissolved in 1 mL H_2_SO_4_ 0.001 N/acetonitrile 95:5 (*v/v*) by using ultrasound and passed through a 0.22 µm nylon filter before analysis. Analyses were performed using a high-performance liquid chromatography system coupled to a diode-array detector (HPLC/DAD, Agilent Technologies, Santa Clara, CA). Chromatographic separation was performed using a LiChrospher 100 RP-18 column (250 × 4 mm; 5 µm; Merck KGaA, Darmstadt, Germany) and a gradient elution program with solvent A) acetonitrile and B) H_2_SO_4_ 0.001 N (flow rate 0.80 mL/min). Chromatograms were recorded at a wavelength of 218 nm. Calibration curves were calculated by analysis of standard solutions at concentrations between 0.022 and 1.3 mg/L prepared by spiking a biological wine for which the absence of phthalimide was previously checked (y = 598924x + 7015; R^2^ = 0.999).

As a precautionary measure, a limit of quantification (LOQ) of 0.001 ppm was adopted for all residues found in the samples, but not for phthalimide. This value is close to the minimum concentration used for calculating the calibration curves, and corresponds to a high signal/noise ratio in wine matrix. For phthalimide, a LOQ of 0.05 ppm was considered. The analytical method used provided the qualitative and quantitative data with high confidence.

## 3. Results

Out of the 17 investigated substances, the analysis performed in the selected samples showed low contents of five (29.4%): cyprodinil, dimethomorph, metalaxyl and zoxamide. In particular, traces of cyprodinil—used against *Botrytis*—were found in all samples (median value 0.001 ppm; interquartile range 0.001; maximum value 0.008 ppm). This concentration is about two orders of magnitude lower than the maximum residue level (MRL) for cyprodinil in wine (0.5 ppm), and three orders of magnitude lower than the MRL registered in Italy on grapes (5 ppm) [30,31,32]. In all samples, traces of dimethomorph (used against downy mildew) were also found (median value 0.012 ppm; interquartile range 0.008). The maximum content was 0.04 ppm, around 10-fold lower than the MRL on grape (0.5 ppm) [31].

Traces of metalaxyl, which is used against downy mildew, were found in all samples (median value 0.044 ppm; interquartile range 0.041). Three samples showed the highest concentrations (0.122, 0.127 and 0.155 ppm respectively) though at values lower than the MRL in wine (0.2 ppm; the MRL on grape is 1 ppm) [31]. Overall, 37 (74%) samples had traces of zoxamide (median value 0.001 ppm; interquartile range 0.001), which is used against downy mildew. In this case, the concentration was again much lower than the MRL in wine (0.5 ppm, 5 ppm on grape) [31].

Ethylene thiourea was never found, but phthalimide was found in eight (16%) samples (median value 0.1 ppm; interquartile range 0.1; maximum value 0.2 ppm). Potentially, this metabolite is a marker to reveal the use of folpet, a pesticide that is not allowed by Conegliano-Valdobbiadene Prosecco DOCG Viticulture Protocol.

**Figure 1:**
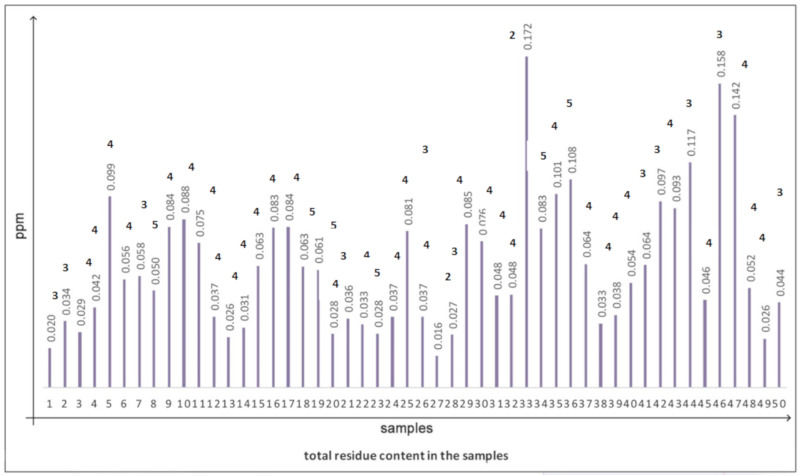
Total pesticide contents calculated as sums of the individual active substances found in the samples. All data are lower the MRL set in Italy for metalaxyl in wine (0.2 ppm, DM 27/08/2004). The number of active principles found in the samples are reported above the bars.

By summing each of the active ingredients found, the total pesticide content was calculated for each of the 50 samples. Data are shown in Figure 1; the maximum value was 0.172 ppm. This value is lower than the MRL of metalaxyl in wine in Italy (0.2 ppm), which is the most restrictive among those of the residues found in the samples.

## 4. Discussion

The present study reports the results of an investigation aimed at detecting the presence of the 15 active substances allowed “just for limited use” by the Conegliano-Valdobbiadene Prosecco Superiore DOCG Viticulture Protocol [19], in addition to phthalimide and ethylene thiourea, in a sample of 50 Prosecco DOCG wine bottles. Legislation states that foods that are not of animal origin can be commercialized only if they contain residues of active substances lower than their MRL [33,34,35]. In our study, the presence of five active substances was detected. In particular, all the samples showed traces of at least two PPP residues, but their contents were much lower the MRLs allowed by the law.

Results of community control plans published by the European Food Safety Authority through scientific reports showed that out of 1315 grape samples collected in 2017 in the member countries, 821 (62.4%) had no residues, 270 had just one active ingredient (20.6%), and 224 showed the presence of more pesticides (17%) [36,37]. Moreover, regarding the specific production process, it seems useful to consider that sector studies have shown that the chemical mechanisms involved in alcoholic fermentation can reduce the concentration of fungicide residues in the final products [38].

Data released in 2019 by Italian Ministry of Health relating to the official controls conducted in the year 2017 by the Competent Authorities, including the Food and Nutrition Hygiene Services of the Local Health Units, show that 732 wine samples have been controlled [39]. For the purposes of official control of pesticide residues in food products of non-animal origin, the sampling techniques must comply with the provisions of the EU Directive 2002/63/EC, implemented in our country with a ministerial decree in 2003 [40,41]. A total absence of residues was detected in 434 samples (59.3%), whereas 298 samples (40.7%) showed the presence of PPP residues below the MRLs, and were therefore considered regular. In 155 of the latter samples just one residue was found, while multiple residues were detected in 143 samples [39].

In our study, all the samples tested were positive for more than one active substance, ranging from two to five substances for each sample. Although the evaluation and health risk assessment for each pesticide taken separately is generally well regulated at the national and international levels, the aspects concerning possible interactions between the active products is still a matter of debate.

Moreover, it should be underlined that people can be exposed to more than one pesticide through the diet on a daily basis, both due to the presence of multiple active substances in one food, and to the simultaneous intake of different foods containing single substances. If these substances have the same toxicological endpoint and the same mechanism of action, the conventional way of assessing the dietary risk of exposure to pesticides separately may lead to an underestimation of the health risk [42]. For this reason, in the extensive discussion on exposure assessment to residues of PPPs provided by the Codex Alimentarius and promoted by the Food and Agriculture Organization and World Health Organization [43], the theoretical maximum daily intake values should be evaluated considering the entire diet and not the individual components. Therefore, it is not sufficient to quantify the presence of individual residues in a single food matrix when aiming to assess the real daily exposure to PPP residues [44].

Our results show low levels of active substances—even when summing the individual active ingredients the maximum value was 0.172 ppm (lower than the most restrictive MRL of metalaxyl). With regard to this, despite some exceptions, it has been demonstrated that interaction between components is a rare event at low levels of exposure. Moreover, recent studies have shown that the chronic cumulative intake of low levels of pesticide residues appears to be relatively well under the ADI (acceptable daily intake), and no long-term consumer risk is expected [45]. Regardless, research should continue to seek more in-depth insight in the complex field of combination toxicology at low doses, and to develop predictive and more efficient testing methods for the risk assessment of chemical mixtures. This should take into account that cumulative exposure is the simultaneous or subsequent exposure to various chemicals that contribute to a cumulative effect [45].

In the official controls performed by the Italian Competent Authorities in 2017, the active substances most frequently found were metalaxyl and dimethomorph [39]; this data is consistent with our results, which show the presence of metalaxyl and dimethomorph residues in all the investigated samples.

Although our findings confirm that PPP residues are present in Prosecco DOCG wines, it must be emphasized that our study was performed using high-resolution mass spectrometry, which is a very sensitive analytical technique [46]. By this method, the signals of residues present at very low levels are recorded.

With regard to phthalimide, its detection in eight Prosecco wine samples cannot be confidently attributed to folpet use on the vineyard because its presence—especially at trace levels—could also be due to wind transport during the treatments carried out in nearby vineyards where the restrictive DOCG protocol is not applied. For these reasons, future efforts have to be targeted to proposing regulations which also account for the origin of accidental contaminations.

In addition to the possible presence of PPP residues detectable in raw materials or finished products, the treatments carried out on extremely permeable agricultural soils, below which groundwaters could flow, can also expose water intended for human consumption to the risk of contamination by active substances [47,48].

Lastly, recent studies highlight the need to deepen the knowledge regarding mechanisms of action and investigations into the durability and bioavailability of biopesticides for a reliable assessment of risks and benefits [49].

The authors are aware that further studies must be performed in order to deepen the investigated aspects in a wider sample. Moreover, additional issues should be explored, such as the influence of PPP residues on alcoholic fermentation, the bioavailability of different PPPs in hydro-alcoholic medium, and risks to consumer health. Lastly, new validated analytical methods should be tested and proposed.

## 5. Conclusions

Residues detected in the investigated samples were always clearly lower than the MRLs, in compliance with sector regulation. Although the study was carried out in only one year, these preliminary findings highlight that the production protocol and control system are sufficiently tight and restrictive to guarantee the production of wines having quality standards that are higher than required by the regulations. Regardless, future efforts should be targeted to further reduce or eliminate the presence of PPPs in these high-quality wines.
